# Dark triad and cyber aggression among Chinese adolescents during COVID-19: A moderated mediation model

**DOI:** 10.3389/fpsyg.2022.1011123

**Published:** 2022-11-21

**Authors:** Zhen Zhang, Shengnan Bian, Hui Zhao, Chunhui Qi

**Affiliations:** ^1^Faculty of Education, Henan Normal University, Xinxiang, China; ^2^Fang Cheng No.1 Senior Middle School, Nanyang, China

**Keywords:** dark triad, cyber aggression, moral disengagement, gender differences, adolescents

## Abstract

During the COVID-19 pandemic, the use of online learning has become a necessary choice for students, and would increase the probability of cyber aggression (CA). Despite the relationship between Dark Triad and CA previous was explored in previous research, the underlying psychological mechanism of CA in adolescents is still unclear. The current study aimed to examine the mediating role of moral disengagement (MD) and the moderating of gender in the relationship between Dark Triad and CA. A sample consists of 501 Chinese adolescents (246 females; 255 males) between the ages of 11 ~ 20. Participants completed the Dirty Dozen Scale, Moral Disengagement Scale, and Cyber Aggressive Behavior Scale. Results show that higher levels of dark personality were associated with higher levels of MD and CA. Moral disengagement partially mediated this positive effects of dark personality on CA. Moreover, gender moderated the mediation model. Specially, the positive relationship between dark triad personality and CA was stronger among females adolescents. These findings advance the understanding of how dark triad personality induces Chinese adolescents’ cyber aggressive behavior.

## Introduction

Due to the rapid expansion of the internet and the popularization of computers and smartphones, cyber aggression (CA) has gradually become an important public health problem with serious implications for adolescents’ social relations, academic performance and mental health (Grigg, 2010; Modecki et al., 2014). CA refers to a new kind of aggressive behavior in which online technology and mobile devices are used to harm others for malicious purposes (Grigg, 2010; Zhang and Zhao, 2020). Whenever one intends to harm an individual or group of individuals through internet, they are engaging in cyber aggressive behavior. Youth can also be victims of CA as well as perpetrators. For example, the number of adolescents who have attacked others on social media was approximately 52% (Festl and Quandt, 2013), and 75% of adolescents reported experiencing CA through their use of the internet (Chapin, 2016). The use of cyber aggressive behavior among teenagers presents a significant threat, not just to the victims, but also to the perpetrators’ mental and physical health. Number of research found that CA is linked to traditional bullying (Modecki et al., 2014), mental health problems (Mishna et al., 2018), substance use (Crane et al., 2021), delinquent behavior (Farrell et al., 2020), and suicidal idearion (Schenk and Fremouw, 2012). As a result, a large number of researchers have been devoted to exploring potential factors that can inhibit cyber aggressive behavior so as to weaken and reduce it.

In numerous disciplines, scholars explore the predictors of CA from a variety of perspectives. The focus of cognitive scientists, psychologists, and behavioral economists is often on individual factors such as negative emotions, self-control and personality trait (Pabian et al., 2015; Zhang and Zhao, 2020), while educationalists and sociologists usually emphasize external social forces, including social circumstances and moral norms (Wright et al., 2020; Bullo and Schulz, 2022). However, research involving Chinese adolescents remain scarce (Zhang and Zhao, 2020; Zhu et al., 2020), partly due to the restricted used of network electronic products. The occurrence of the COVID-19 pandemic has changed this status quo. Chinese children and adolescents have to learn online and interact with teachers and classmates through the Internet, which might significantly increase CA in adolescents. Hence, it is urgent to explore the potential influential factors of CA in order to reduce its negative impacts.

## Literature review and research hypotheses

### Dark triad and cyber aggressive behavior

Cyber aggression involves intentional damage delivered *via* digital means to another person or persons (Corcoran et al., 2015), including online stalking, harassment, flaming, use of profanities and group exclusion (Mladenović et al., 2021). Youth, in particular, are adversely affected by CA, which makes it an urgent issue for school districts, communities, and governments. According to the personality process model of CA (Gammon et al., 2011), the Dark Triad (DT) is an important predictor variable influencing CA. The dark triad consists of three interrelated characteristics, namely, Machiavellianism, psychopathy, and narcissism (Paulhus and Williams, 2002). Specifically, Machiavellianism is characterized by disregarding moral principles in order to accomplish goals (Jones and Paulhus, 2010); psychopathy is a personality trait represented by impulsivity, lack of responsibility and empathy (Patrick et al., 2009); narcissism is a normal and continuously distributed personality trait, which is often characterized by extreme arrogance, superiority, privilege and deprivation (Krizan and Herlache, 2018).These traits are characterized by some common features, e.g., violating social values (Kajonius et al., 2015), social aversive callousness (Jones and Paulhus, 2010), a fast and exploitive life history strategy (Jonason and Webster, 2010), reduce empathy (Heym et al., 2019), disagreeableness, and impulsivity (Paulhus and Williams, 2002).

In general, the dark triad is associated with a wide variety of negative problems, such as antisocial behaviors (Sijtsema et al., 2019), delinquency (Alsheikh Ali, 2020), internet addiction (Lee and Lim, 2021), aggressive behaviors (Zhu and Jin, 2021), and cheating behavior (Nicholls et al., 2020). Moreover, number of studies have demonstrated that adolescents’ dark triad traits are positively correlated with a wind range of aggression in real life, such as physical and verbal aggression (Jones and Neria, 2015), reactive/proactive aggression (Dinić and Wertag, 2018), relational aggression (Erzi, 2020), bullying (Davis et al., 2022), and driving aggression (Ball et al., 2018). In addition, previous empirical research also indicated that dark triad traits significantly predict adolescents’ CA (Moor and Anderson, 2019; Zhang and Zhao, 2020; March and Marrington, 2021). Therefore, based on the above, current research highlights dark personality as an predisposing risk factor that significantly predicts potential cyber aggressive behavior. Based on the above analysis, we propose the following hypothesis.

Hypothesis 1: High DT could result in higher adolescents’ CA during the COVID-19 pandemic.

### Mediating effect of moral disengagement

In previous studies, it has been noted that dark triad personality traits in adolescents correlate with their CA (Pabian et al., 2015; Moor and Anderson, 2019), but the potential psychological mechanisms involved have never been fully understood. Moral disengagement (MD) is one potential explanation for the proposed influence of dark triad characteristics on adolescents’ CA. MD is characterized by eight different cognitive mechanisms that enable individuals to violate internalized moral norms and to act unethically without guilt (Moore, 2015). According to social cognitive theory (SCT, Bandura 1989), individuals usually act in prosocial ways and avoid antisocial behaviors due to internal standards. However, people could adopt MD to rationalize their immorally acts, thereby reducing their negative self-judgment and guilty feelings (Bandura et al., 1996). Following this reasoning, numerous empirical and review studies had found that MD promotes various unethical behaviors in adolescents, including aggression, bullying, and cyber bullying (Killer et al., 2019; Bjärehed et al., 2020; Falla et al., 2021; Lo Cricchio et al., 2021). For example, a systematic review found that MD was significantly related to cyber bullying even after the roles of moderating variables were controlled (Lo Cricchio et al., 2021). Thus, MD in adolescents would be speculated as positively associated with their CA.

In additional, based on the life history theory (McDonald et al., 2012), the common characteristics of Dark triad, including egoism, violating social values, and emotional coldness, might enhance promote the possibility of justifying immoral consequences through MD (Sijtsema et al., 2019). Some research have demonstrated that Dark Triad personality is associated with MD (Egan et al., 2015; Sijtsema et al., 2019; Erzi, 2020; Kapoor et al., 2021). Therefore, it is expected that the Dark triad personality will has a positive relationship with MD. In summary, it is hypothesized that adolescents with a higher Dark triad personality trait tend to rely more on MD strategies, which then could promotes their cyber saggression during the COVID-19 pandemic. In light of the above analysis, we develop the second hypothesis.

Hypothesis 2: MD could mediate the association between DT and adolescents’ *CA*.

### Moderating effect of gender

Gender is another important factor of interest for our research. Social role theory (SRT, Eagly and Wood, 1999) argues than men and women were assigned different gender roles and stereotypes, which make females act in a more selfless and communal oriented manner than males in a variety of social situations. Consequently, empirical and meta-analytical studies have found that males consistently reported higher scores on dark triad personality (Jones and Weiser, 2014; Muris et al., 2017). In addition, studies on CA indicated that women are prone to engage in relational and indirect CA, while men engage in more direct and physical CA (Carbone-Lopez et al., 2010; Nocera and Dahlen, 2020). Moreover, it might be that males are more prone to involvement in cyber aggressive behaviors than females, because of their higher levels of dark personality features.

In reality, findings regarding gender differences in relations between dark triad and CA are limited and inconclusive. Some researcher found that Machiavellianism (Kircaburun et al., 2018) and grandiose manipulative trait (a sub construct of psychopathy; Ciucci et al., 2014; Orue et al., 2016) better predict CA among men, whereas others indicated that callous-unemotional trait (another sub construct of psychopathy; Orue et al., 2016) and psychopathy (Zhu and Jin, 2021) was more strongly related to aggression in girls. However, other studies shown that gender could not moderate these relationship (Nocera and Dahlen, 2020; Wright et al., 2020). Thus, it becomes essential for examining the gender differences between these variables. Taken together, according to SRT and previous findings, we hypothesize that Dark triad personality are more strongly associated with CA for males than females. Therefore, we further propose the third hypothesis.

Hypothesis 3: DT would be more strongly associated with CA for males than females.

**Figure 1:**
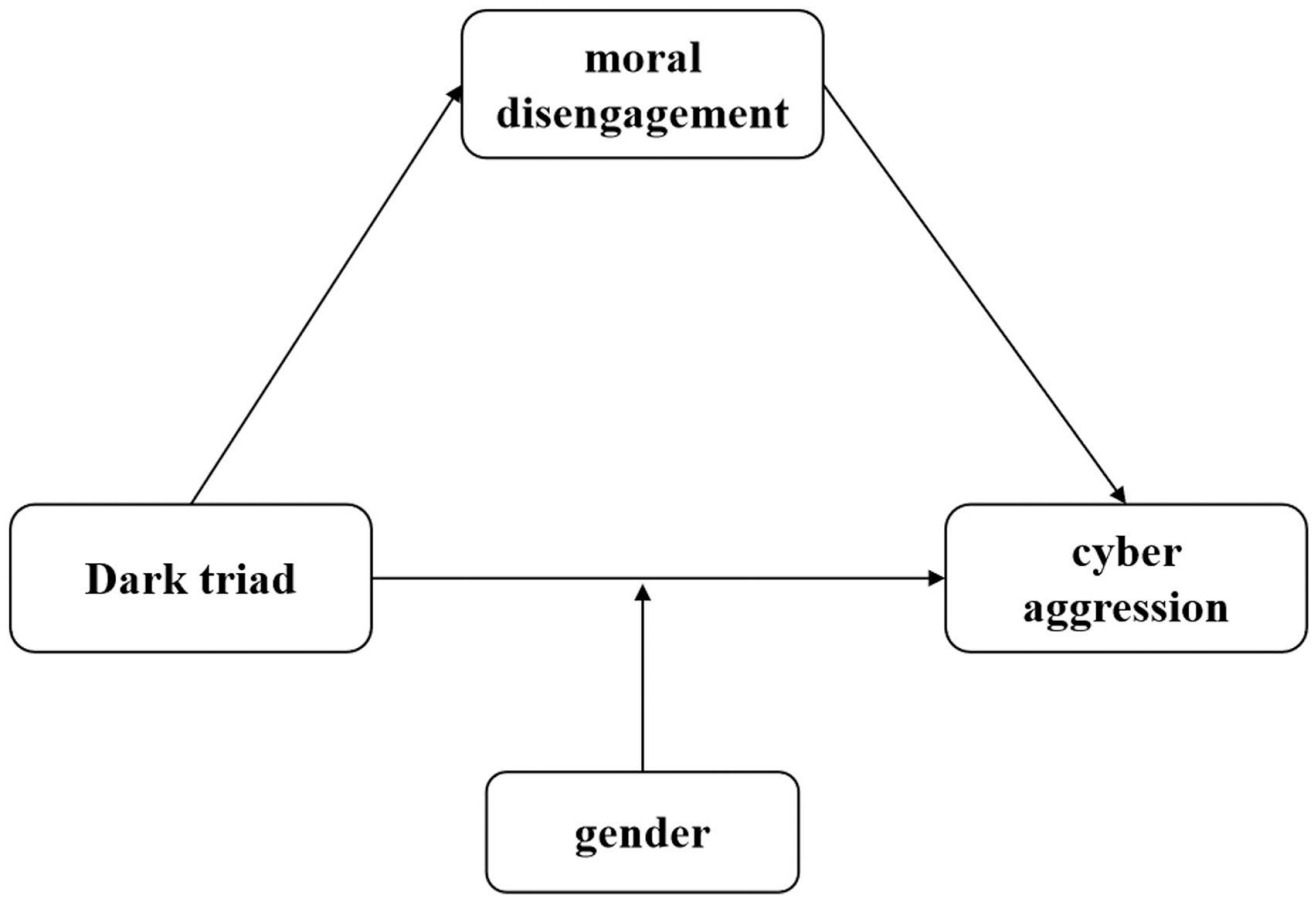
Research model.

To sum up, based on social cognitive theory and social role theory, the intention of present study is to investigate a moderated mediation model to outlines the mechanism underlying the connection between DT and Chinese adolescents’ CA during the COVID-19 pandemic. Figure 1 depicts the research model.

## Materials and methods

### Participants and procedure

As a method of testing the proposed model, questionnaire survey method was used in this study to collect research data. The data are mainly from junior high school students in Henan Province, China. An experienced research assistant distributed and gathered the survey data, and all aspects of the survey process were standardized. Students in one middle school were surveyed over a 2-week period in October 2020. Approval for this study was obtained from the ethics committee of the Faculty of Education, Henan Normal University.

Our survey consisted of three questionnaires. A number of commonly used control variables were also added to the study, such as age, gender, grade, etc. Data were collected in a voluntary and anonymous manner to prevent societal expectations and response biases. Specifically, we informed participants that all data would be used for scientific research and would not be linked to their personality traits or academic assessments. At any time, they can leave if they feel uncomfortable. A total of 600 questionnaires were distributed to students who took a group test, and 521 questionnaires were collected, with an initial recovery rate of 86.83%. As a result of suspicious responses and missing values, 20 of the 521 cases were removed. The effective response rate was 83.50% after removing 20 cases.

The final valid sample consisted of 255 boys (50.90%) and 246 girls (49.10%) between the ages of 11 and 20 (*M* = 14.01, *SD* = 1.07). Among them, 87 (17.37%) were in the seventh grade, 206 (41.12%) were in the eighth grade, and 208 (41.51%) were in the ninth grade. With the help of a well-trained research assistant, participants completed the measures voluntarily in regular schoolrooms. Subjects were required to provide written or oral informed consent. The research lasted approximately 25 min.

### Measures

#### Dark triad

Adolescents’ dark personality characteristics was evaluate by adopting the 12 items version of the Dirty Dozen Scale (DDS, Jonason and Webster, 2010). Each characteristic contains four items: Machiavellianism (e.g., “I have use flattery to get my way.”), psychopathy (e.g., “I tend to be cynical.”) and narcissism (e.g., “I tend to want others to admire me.”). The Chinese version had been validated by Geng et al. (2015), and was used in this study. Participants assessed each item on a seven point scale ranging from 1 (strongly disagree) to 7 (strongly agree), with higher average score on the subscale reflecting higher dark personality. The Cronbach’s *α* of Machiavellianism, psychopathy and narcissism were 0.83, 0.70 and 0.84 in present study, respectively.

#### Moral disengagement

Adolescents’ MD was assessed by adopting the 8 items version of Moral Disengagement Scale (MDS, Moore et al., 2012). Questions for example, “Taking something without the owner’s permission is okay as long as you are just borrowing it.” The Chinese version has been validated (Zheng et al., 2019) and was used in the present study. Subjects was asked to rate the items on a five point scale (1 = totally disagree, 5 = totally agree), with higher the overall mean score implying a stronger level of MD. The Cronbach’s *α* coefficient of this scale is 0.88 in this study.

#### Cyber aggression

The Cyber Aggressive Behavior Scale, developed by Zhao and Gao (2012) in China, was used to explore adolescents’ CA. This scale consists of 31 items and two subscales: instrumental aggression and reactive aggression. This study only uses the instrumental aggression subscale which contains 15 items, e.g., “In order to get the results I want, I often insult and scold others when playing online games.” All items were scored on a four-point scale (1 = never, 4 = always) by the participants, with higher the overall mean score reflecting a stronger level of cyber aggressive behavior. The Cronbach’s α coefficient of this scale with the current sample is 0.91.

### Data analysis

All data analysis were completed in SPSS 26.0 and Process macro developed by Hayes (2017). The first step was to calculate descriptive statistics for all variables and to perform correlation analysis on them. The second step was to examine the mediated role of MD by applying Model 4. After that, Model 5 was examined to determine whether gender could moderate the indirect path between MD and adolescents’ *CA*. All study variables were standardized, and the bias-corrected bootstrapping method with 5,000 samples was conducted.

## Results

### Descriptive statistics and correlation analysis

The descriptive statistics are reported in Table 1. Gender differences were explored, and males scored significantly higher than females on the dark triad personality, MD, and CA scores. Correlational analysis is reported in Table 2. The results found that dark triad traits were positively related to MD and *CA*. A negative correlation was found between MD and *CA*. These results implied that individuals with a dark personality have stronger CA, which supports Hypothesis 1.

**Table 1:** Descriptive statistics according to gender and *t*-test scores.

	Female (*N* = 246)		Male (*N* = 255)		*t*
	*M*	*SD*	*M*	*SD*	
Machiavellianism	1.48	0.79	1.91	1.27	−4.57**
Psychopathy	1.83	0.91	2.14	1.18	−3.29**
Narcissism	2.74	1.44	3.29	1.65	−4.01**
Moral disengagement	1.47	0.60	1.77	0.79	−4.79**
Cyber aggression	1.16	0.34	1.24	0.36	−2.74**

**p < 0.01.

**Table 2:** Bivariate correlations matrix of all variables (*N* = 501).

	1	2	3	4	5
1. Machiavellianism	1.00				
2. Psychopathy	0.54**	1.00			
3. Narcissism	0.38**	0.40**	1.00		
4. Moral disengagement	0.35**	0.31**	0.28**	1.00	
5. Cyber aggression	0.32**	0.31*	0.22**	0.32**	1.00

*p < 0.05, **p < 0.01.

### Mediation effect analysis

To determine the mediating role of MD between DT and CA, mediation analysis was conducted by using Model 4. Gender and age were included as control variables to reduce potential confounding effects. A summary of the results was shown in Table 3. First, Machiavellianism had positive correlations with MD (β  = 0.32, *p* < 0.001), which in turn was positively related to CA (β = 0.23, *p <* 0.001). Machiavellianism and CA were still significantly connected in a positive way (β = 0.31, *p <* 0.001). Thus, Machiavellianism and CA are linked partly through MD (*b* = 0.07, 95% CI = [0.03 0.12]). In total, MD mediated 23.49% of the impact. Second, psychopathy was positively linked to MD (β = 0.28, *p* < 0.001), which was, in turn, positively linked with CA (β = 0.23, *p* < 0.001). The direct path between psychopathy and CA remained significant (β = 0.29, *p* < 0.001). Hence, psychopathy and CA were associated partly through MD (*b* = 0.07, 95% CI = [0.03 0.11]). MD is responsible for 23.49 percent of the total impact. Third, narcissism positively predicted MD (β = 0.22, *p* < 0.001), which in turn positively predicted CA (β = 0.27, *p* < 0.001). There was a significant direct relationship between narcissism and CA (β = 0.19, *p* < 0.001). Therefore, MD mediated the relationship between narcissism and CA (*b* = 0.06, 95% CI = [0.03 0.10]). The mediation effect of MD was responsible for 30.99% of the influence. In summary, these findings indicated that adolescents’ MD mediates the connection between DT and *CA*. In light of this, Hypothesis 2 was confirmed.

**Table 3:** Testing the mediation effect of moral disengagement (*N* = 501).

Variables	Model 1 (CA)		Model 2 (MD)		Model 3 (CA)	
	β	*t*	β	*t*	β	*t*
Machiavellianism	0.31	7.12**	0.32	7.54**	0.23	5.29**
MD					0.23	5.06**
*R* ^2^	0.34		0.39		0.40	
*F*	21.69**		29.11**		23.48**	
Psychopathy	0.29	6.85**	0.28	6.58**	0.23	5.24**
MD					0.24	5.36**
*R* ^2^	0.33		0.36		0.40	
*F*	20.40**		24.31**		23.33**	
Narcissism	0.19	4.40**	0.22	5.13**	0.13	3.06**
MD					0.27	6.14**
*R* ^2^	0.25		0.32		0.36	
*F*	10.96**		18.35**		18.25**	

MD, moral disengagement; CA, cyber aggression. **p < 0.01.

### Moderated mediation effect analysis

**Figure 2:**
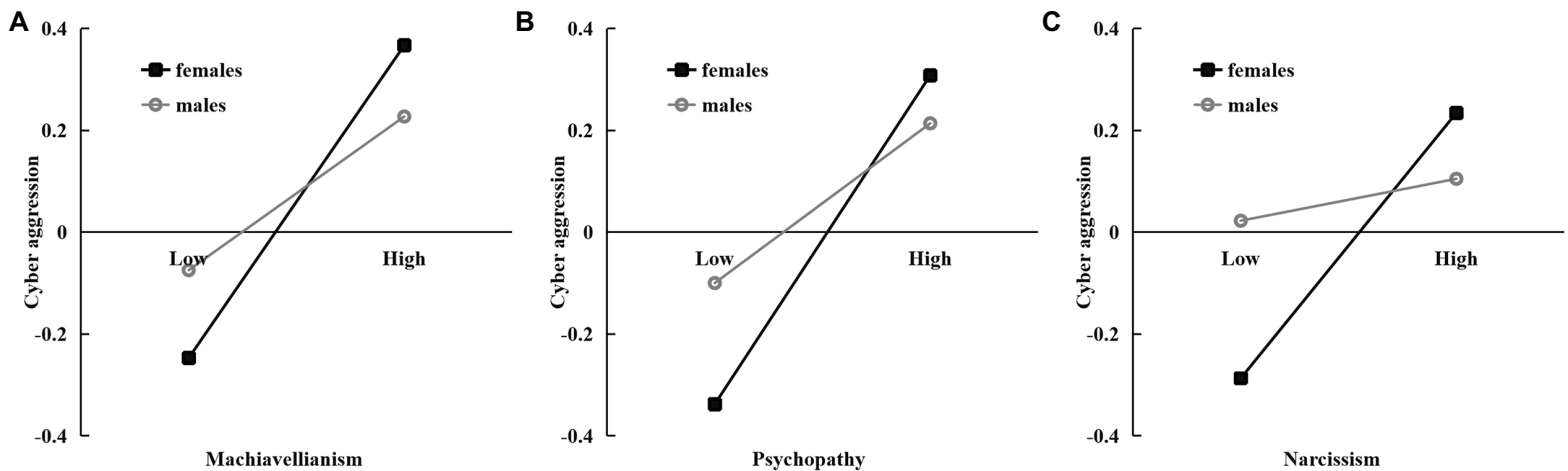
The moderating role of gender in the relation between dark triad personality, i.e., Machiavellianism **(A)**, psychopathy **(B),** and narcissism and cyber aggression **(C)**.

Hypothesis 3 implied that gender might moderate the direct impact of DT on *CA*. Model 5 of the Process macro was adopted to examine this hypothesis. A summary of this results was shown in Table 4. Model 2 of Table 4 shown that the interaction between Machiavellianism and gender had a significantly negative association with CA (β = −0.10, *p* < 0.05). The slope test indicated that the influence of Machiavellianism on CA was stronger in females (β = 0.37, *p* < 0.001) than males (β = 0.18, *p* < 0.001; see Figure 2A). Moreover, the interaction between psychopathy and gender also had a significantly negative relationship with CA (β = −0.09, *p* < 0.05). The slope test implied that psychopathy was more effective in causing CA in women compared to males (β = 0.34, *p* < 0.001) than males (β = 0.16, *p* < 0.01; see Figure 2B). Finally, the interaction between narcissism and gender had a significantly negative connection to CA (β = −0.11, *p* < 0.05). The slope test indicated that the influence of narcissism on CA was stronger in females (β = 0.26, *p* < 0.001) than males (β = 0.04, *p* > 0.05; see Figure 2C). Because gender moderates the direct association between adolescents’ DT and CA, Hypothesis 3 was verified.

**Table 4:** Testing the moderated mediation model (*N* = 501).

Variables	Model 1 (MD)			Model 2 (CA)		
	β	*t*	95% CI	β	*t*	95% CI
Machiavellianism	0.35	8.36**	[0.268–0.432]	0.27	5.66**	[0.181–0.373]
MD				0.23	5.09**	[0.139–0.314]
Gender				0.03	0.58	[−0.059–0.110]
Machiavellianism × Gender				−0.10	−2.01*	[−0.188–0.002]
*R* ^2^	0.36			0.41		
*F*	36.23**			19.71**		
Psychopathy	0.31	7.19**	[0.223–0.391]	0.25	5.55**	[0.160–0.335]
MD				0.25	5.58**	[0.161–0.335]
Gender				0.04	0.92	[−0.045–0.123]
Psychopathy × Gender				−0.09	−1.98*	[−0.172–0.001]
*R* ^2^	0.31			0.41		
*F*	27.17**			19.56**		
Narcissism	0.25	5.88**	[0.170–0.340]	0.15	3.39**	[0.063–0.235]
MD				0.27	6.21**	[0.187–0.359]
Gender				0.05	1.04	[−0.040–0.130]
Narcissism × Gender				−0.11	−2.56*	[−0.194–0.025]
*R* ^2^	0.26			0.37		
*F*	18.56**			16.07**		

Gender was coded as binary variable (0 = female and 1 = male). MD, moral disengagement; CA, cyber aggression. **p < 0.01.

## Discussion

The current study showed that dark triad were positively related to CA among Chinese adolescents, which supported Hypothesis 1. A increasing number of studies have shown that adolescents with high dark personalities tend to engage in aggression, CA, bullying, and antisocial behavior (Moor and Anderson, 2019; Zhang and Zhao, 2020; Zhu and Jin, 2021). In line with these previous studies, these findings observed that all subsets of dark triad personality could significantly predict adolescents’ CA, which might be attributed to their common evil, malevolent and callous features (Book et al., 2015; Muris et al., 2017).

Consistent with Hypothesis 2, the findings showed that MD mediated the connection between all subsets of the dark triad personality and adolescents’ CA. In other words, adolescents high in dark triad personality are more prone to justify immoral consequences, which consequently leads to a rise in cyberattacks. Researchers have previously found that dark triad personality shares some common characteristics, including egoism, violating social values, and emotional coldness, which might enhance the possibility of justifying immoral consequences through MD (Erzi, 2020; Kapoor et al., 2021). In line with these results in Western culture, this study found that dark personality is positively associated with MD among Chinese adolescents. Moreover, adolescents’ MD was positively correlated with CA. Previous research has indicated that MD promotes various unethical behaviors in adolescents, including aggression, bullying, and cyber bullying (Bjärehed et al., 2020; Falla et al., 2021; Lo Cricchio et al., 2021). Consistent with these previous results, our findings supports social cognitive theory (Bandura, 1989) in Chinese culture. In summary, such findings revealed that MD plays a partial mediating role in the association between all dimensions of the dark personality and CA.

Another outstanding contribution of the current study was that gender moderated the association between all subsets of the dark triad personality and adolescents’ CA. These findings help clarify patterns of gender differences in linking dark personality with adolescents’ CA. Contrary to Hypothesis 3, it was observed that all subsets of dark triad personality traits are more strongly associated with CA for women than for men. Most previous studies have demonstrated that Machiavellianism (Kircaburun et al., 2018) and grandiose manipulative trait (a sub construct of psychopathy; Ciucci et al., 2014; Orue et al., 2016) better predict CA among men. In contrast to these findings, this research found that a females’ dark personality was more strongly related to CA. Previous studies found that gender differences in CA were influenced by adolescents’ age, gender stereotype, and the types of behaviors. Wright (2020) found that adolescents who display more feminine characteristics engaged in more cyber relational aggression through social networks and mobile devices. Hence, one potential explanation is that traditional Chinese women have stronger feminine traits, which might, in turn, enhance the effect of dark personality on CA. Furthermore, Barlett and Coyne (2014) indicated that age can modulate gender differences in cyberbullying, with females reporting more cyberbullying during early adolescence. Thus, another possibility could be that the sample was drawn from the early adolescent age range, which might further reinforce the connection between dark personality and girls’ CA. More research needs to be conducted to examine the interaction of dark triad traits, gender and/or gender stereotypes on CA in different age ranges and types of CA.

## Implications of the study

Moderated mediation models, not only reveal the cognitive mechanisms by which dark triad personality leads to cyber aggressive behavior (the mediating role of MD), but also shed light on the underlying individual differences (the moderating role of gender). These results answer the question of how dark triad personality induces aggressive behavior in junior high school students. The study also clarifies the question of among which group of people the direct predictive impact of dark triad personality on cyber aggressive behavior and the indirect effect of MD are more prominent. Therefore, training to weaken MD and effectively prevent and intervene with campus attacks contributes to building a harmonious campus and educational inspiration.

First, moral education work in junior high schools should focus on weakening dark triad personality consciousness. Through the promotion of traditional Chinese mean culture, the demonstration of collectivism, and the training of appropriate MD, students should be motivated to understand themselves objectively and accurately. This proper self-awareness avoids the negative results of excessive selfishness, thereby, effectively reducing campus aggression. Second, schools should provide effective moral attribution training to reduce the MD ability of junior high school students, and thereby realize the model and institutionalization of moral attribution training. Finally, in the process of moral education, educators should focus on dark triad personality intervention and moral attribution training for girls, thereby attenuating their moral detachment and reducing the occurrence of aggressive behavior.

## Limitations and future research

Similar to other studies, this study has various limitations. First, this research used a cross-sectional design, which might restrict its ability to determine causality. Further study should consider the use of longitudinal or experimental designs to confirm causal relations. Second, given that the subjects were recruited from the same junior middle school in China, the extent to which the findings generalize to other age groups is restricted. Future studies could recruit a sample representing the full age range and explore whether age moderates the proposed patterns. Finally, multiple factors (e.g., empathy and emotional intelligence) may also affect the link between dark triad personality and CA. Future studies might try to incorporate more variables.

## Conclusion

The present study was conducted to examine the psychological mechanisms that might underlie CA in Chinese adolescents, which promoted a better understanding of the association between dark triad personality and CA. The results of present study shown that (1) The dark triad personality subtypes are all significantly positive for CA; (2) MD mediates the connection between all subsets of the dark triad and CA among adolescents; and (3) all subsets of dark triad personality are more effective in causing CA in women compared to males.

As a result of these findings, the literature on CA on university campuses is enriched in important ways. These results also contribute to a growing understanding of how dark triad personalities results in CA. Moreover, we revealed that that MD, as a set of cognitive strategies, can enhance the positive effect of dark triad personality on CA. All of these conclusions imply that school administrators and teachers should prevent overuse of MD strategies when designing psychological interventions to reduce CA, especially for girls.

## Data availability statement

The raw data supporting the conclusions of this article will be made available by the authors, without undue reservation.

## Ethics statement

The studies involving human participants were reviewed and approved by the University Ethics Committee at the Henan Normal University. Written informed consent to participate in this study was provided by the participants’ legal guardian/next of kin.

## Author contributions

CQ and ZZ designed the experiment. HZ and SB collected and analyzed the data. CQ, SB, and ZZ wrote the manuscript. All authors contributed to the article and approved the submitted version.

## Funding

This study was supported by the National Natural Science Foundation of China (No. 32000754), Youth Foundation of the Ministry of Education of Humanities and Social Science Project of China (No. 20YJC190030), Science and Technology Research Project of Henan Province (No. 222102320386) and Philosophy and Social Science Foundation of Henan Province of China (Nos. 2019CJY030 and 2021CJY052).

## Conflict of interest

The authors declare that the research was conducted in the absence of any commercial or financial relationships that could be construed as a potential conflict of interest.

## Publisher’s note

All claims expressed in this article are solely those of the authors and do not necessarily represent those of their affiliated organizations, or those of the publisher, the editors and the reviewers. Any product that may be evaluated in this article, or claim that may be made by its manufacturer, is not guaranteed or endorsed by the publisher.
